# Beliefs About Transitional Events: The Effect of Experience and Life-Script Consistency

**DOI:** 10.3389/fpsyg.2021.727524

**Published:** 2021-08-11

**Authors:** Liangzi Shi, Norman R. Brown

**Affiliations:** ^1^Department of Psychology, University of Alberta, Edmonton, AB, Canada; ^2^College of New Caledonia, Prince George, BC, Canada

**Keywords:** transition theory, cultural life script, autobiographical memory, autobiographical belief, life transition

## Abstract

The present study examined the beliefs about two types of important life transitions: transitions that are consistent with the cultural life script (e.g., getting married) and transitions that diverge from it (e.g., relocating). Data were collected from two conditions: individuals in the experienced condition only responded to transitions they had experienced; individuals in the hypothetical condition provided ratings only for transitions they had not experienced. Participants rated the likelihood and typical age of occurrence, importance, transitional impact, and valence for an individualized set of condition-appropriate events. We found that script-consistent events were considered more normative and positive than script-divergent events. The two types of events, however, differed little in terms of importance or transitional impact. We conclude by arguing that although script-consistent and script-divergent transitions have much in common from a mnemonic perspective, the distinction is still warranted in the context of lifetime planning and evaluation.

## Introduction

*Transitions* are events that cause fundamental and enduring changes to the “fabric of daily life” (Hareven and Masoka, 1988; Shum, 1998; Brown et al., 2012, 2016; Brown, 2016, 2021). Currently, transitions are being investigated from two quite different angles. One line of research has focused on the *cultural life script*. According to Berntsen and Rubin (2004) “a life script represents a series of events that take place in a specific order and represent a prototypical life course within a certain culture… [Included] in life scripts… are culturally *important transitional events* that are expected to occur within a circumscribed age span in the life course of individual members of the culture” (p. 429, emphasis added). These authors also assert that “cultural age norms, prescribing an appropriate age for the event in question” (p. 429) are the most important criterion for inclusion in the life script. In general, this line of research has been aimed at identifying the set of events that belong to the cultural life script (Berntsen and Rubin, 2004; Dunlop et al., 2017; Grysman and Dimakis, 2018), documenting cross-cultural similarities and differences in life-script membership (Erdogan et al., 2008; Rubin et al., 2009; Zaragoza Scherman, 2013; Janssen et al., 2014; Zaragoza Scherman et al., 2017; Janssen and Haque, 2018; Özbek et al., 2021), and demonstrating the importance of *script-consistent* transitions in organizing autobiographical memory (Berntsen and Rubin, 2004; Habermas, 2007; Thomsen and Berntsen, 2008; Bohn, 2010; Dickson et al., 2011; Janssen and Rubin, 2011; Thomsen et al., 2011; Umanath and Berntsen, 2013; Alea et al., 2014; Ece and Gülgöz, 2014; Bohn and Habermas, 2016; Janssen and Haque, 2018).

In brief, “only events considered to be important, expected [age-normative] transitional events … are included in the life script” (p. 429). Berntsen and Rubin (2004), however, also recognize that “some events may be highly *personally* significant without fulfilling these requirements (e.g., a serious accident, winning the lottery). Such events would be associated with no age norms” (p. 429, emphasis in the original). Interestingly, such *script-divergent* events (also known as *nonnormative events*, Baltes et al., 1980) are at the center of a second line of transition-focused research, research that has established the existence of *collective transitions* (e.g., wars, natural disasters). This line of investigation has demonstrated that both collective transitions and individually-experienced script-divergent transitions (e.g., immigration, relocation) have a strong predictable effect on the contents and the structure of autobiographical memory (Schrauf and Rubin, 2001; Brown et al., 2009, 2012, 2016; Brown and Lee, 2010; Zebian and Brown, 2014; Nourkova and Brown, 2015; Uzer and Brown, 2015; Bohn and Habermas, 2016; Enz et al., 2016; Shi and Brown, 2016; Gu et al., 2017, 2019; Camia et al., 2019).

These findings, in turn, have come to underpin *Transition Theory* (Brown et al., 2012, 2016; Brown, 2016, 2021). Among other things, Transition Theory assumes that the mnemonic importance of a particular transition reflects its impact (i.e., the magnitude of the change it has brought about), independent of its normativity (script-consistence vs. script-divergence) or its scope (collective vs. personal). In other words, on this view, from a functional perspective, it does not matter whether a given event is part of the cultural life script. Instead, what is important is how much change has followed in its wake. Consistent with this point, both script-consistent transitions and script-divergent ones are frequently used as temporal landmarks (e.g., Bohn and Habermas, 2016; Shi and Brown, 2016; Gu et al., 2017; Camia et al., 2019) and give rise to a relatively large number of memorable personal experiences (Pillemer et al., 1986, 1988; Kurbat et al., 1998; Thomsen and Berntsen, 2005; Uzer and Brown, 2015; Brown et al., 2016; Enz et al., 2016; Shi and Brown, 2016; Gu et al., 2017; Thomsen et al., 2021). In addition, both types of events are often mentioned when participants are asked to recall important events from their own lives (Glück and Bluck, 2007; Thomsen and Berntsen, 2008; Rubin et al., 2009; Bohn, 2010; Haque and Hasking, 2010; Dickson et al., 2011; Thomsen et al., 2011; Gu et al., 2017, 2019) and from the lives of their parents (Svob and Brown, 2012; Svob et al., 2016; Gu et al., 2019).

On this view, then, what distinguishes script-consistent transitions from script-divergent ones is not the way they affect autobiographical memory. Rather, the main differences are: (a) the knowledge that restrictive age norms are associated only with script-consistent events (Greene, 1990; Greene et al., 1992; Settersten and Hagestad, 1996a,b; Berntsen and Rubin, 2004; Habermas, 2007; Rubin et al., 2009; Thomsen et al., 2011; Alea et al., 2014; Grysman and Dimakis, 2018); (b) the tendency for script-consistent events to elicit higher prevalence estimates than script-divergent events^1^ (Greene, 1990; Berntsen and Rubin, 2004; Habermas, 2007; Rubin et al., 2009; Umanath and Berntsen, 2013; Bohn and Habermas, 2016; Enz and Talarico, 2016; Grysman and Dimakis, 2018); and (c) the fact that script-consistent transitions are typically positively valenced and script-divergent ones are not (Erdogan et al., 2008; Dickson et al., 2011; Thomsen et al., 2011; Umanath and Berntsen, 2013; Grysman and Dimakis, 2018; Janssen and Haque, 2018). In addition, script-divergent events have been shown to be related to mental or somatic illness (Theorell, 1976; Bloom et al., 1978; Sarason et al., 1978).

Transition Theory assumes that it is the transitional nature of script-consistent and script-divergent events that is central to our understanding of them and to their mnemonic importance. The temporal normativity and high prevalence may primarily reflect the importance of script-consistent transitions in a sociocultural sense, rather than their role in the organization of autobiographical memory. Motivated by this implication, the present study was designed, in part, to measure the transitional nature of script-consistent and script-divergent events as well as their importance, valence, likely age of occurrence and likelihood of occurrence. In addition, for reasons developed below, this study allowed us to examine the effect of experience on these judgements.

The experiment itself involved data collection from 247 individuals aged from 18 to 25 (younger group) and 50 to 65 (older group); half were assigned to an *experienced* condition and half to a *hypothetical* condition. During the first phase, all participants were presented with a list of thirty event descriptions; half described common script-consistent events, and half common script-divergent events. During this phase, individuals indicated whether they had experienced the event in question. This phase provided information about the prevalence of the listed events and enabled us to select a participant-specific set of experienced and not-experienced events. We knew that script-consistent events, but not script-divergent ones, tended to be experienced during late adolescence and early adulthood (Greene, 1990; Greene et al., 1992; Settersten and Hagestad, 1996a,b; Berntsen and Rubin, 2004; Habermas, 2007; Rubin et al., 2009; Thomsen et al., 2011; Umanath and Berntsen, 2013; Alea et al., 2014; Bohn and Habermas, 2016; Enz and Talarico, 2016; Grysman and Dimakis, 2018). Thus, we included older participants so that we could collect a reasonable amount of data for experienced events.

Following this initial phase, participants were presented with (up to) eight script-consistent events and (up to) eight script-divergent events. In the experienced condition, the target events were drawn at random from the set of “experienced” events; in the hypothetical condition, they were drawn at random from the set of “not experienced” events. In both conditions, participants were asked to estimate the prevalence and likely age of each event. As is typical of research in this area, participants in the hypothetical condition were instructed to consider the event in question from the perspective of an average Canadian, and then to rate it in terms of its likely importance and valence and to indicate its probable transitional impact. In contrast, in the experienced condition, participants provided a brief description for the experienced event and then rated that event in terms of its *actual* importance, its *actual* valence and its *actual* transitional impact. Thus, this design allowed us to compare beliefs about script-consistent and script-divergent events and to assess the degree to which subjective beliefs and real-world experience aligned.

Focusing on the hypothetical condition, we expected to replicate the usual differences between script-consistent and script-divergent events. Thus, we predicted that: (a) script-consistent events would be rated as being more positive than script-divergent events, (b) script-consistent events would elicit higher likelihood ratings than script-divergent events, and (c) age estimates provided for script-divergent events would be more temporally normative (i.e., be less variable) than those provided for script-divergent events. Although we expected that some of these events might be judged to be more important and more transitionally impactful than the others, we predicted that (d) these differences would not be systematically related to script status (i.e., script-consistent vs. script-divergent).

We also expected that (e) participants in the experienced condition would also give higher likelihood ratings to script-consistent events than to the script-divergent ones and that (f) script-consistent events would elicit less variable age estimates than script-divergent events. For these measures, then, the predictions were the same for both the experienced and hypothetical conditions. However, we also had reason to believe that (g) participants in the experienced condition would provide higher likelihood ratings overall. Specifically, we know that people often rely on the *availability heuristic* when estimating event frequencies and probabilities (Tversky and Kahneman, 1973; Carroll, 1978; Lichtenstein et al., 1978; Weinstein, 1980; Fitzgerald et al., 1988; MacLeod and Campbell, 1992; Brown, 2002). If availability plays a role when people generate these estimates and if event knowledge is more readily available when an event has been experienced than when it has not, participants in the experienced condition should provide higher likelihood ratings.

As noted above, the present design made it possible for us to compare people's beliefs about the importance, transitional impact, and valence of a set of potentially significant personal events with ratings provided by people who have had actual experience with those events. At the outset, we expected that (h) participants in the hypothetical condition would provide higher ratings on these measures than those in the experienced condition and that (i) this would be true for both script-consistent and script-divergent events. There was good empirical support for this prediction. Specifically, a number of studies have examined specific life transitions (e.g., the transition to university, Lauterbach and Vielhaber, 1966; Stern, 1966; Buckley, 1971; King and Walsh, 1972; the birth of a first child, Belsky, 1985; Luhmann et al., 2012). The findings suggest a mismatch between people's expectations and their experience; apparently, people tend to overestimate the long-term impact of important life events such as entering university. We expected to replicate this bias using a wide set of events.

Although people might have somewhat unrealistic expectations about the consequences of important life events, they may still have an accurate understanding of their relative importance. In addition, people may well-recognize that script-divergent events can be just as impactful as script-consistent events. These intuitions led us to predict the following: Using events as the unit of analysis, (j) mean importance, transitional impact, and valence ratings collected from the hypothetical condition should correlate highly with those collected from the experienced condition; and overall, (k) there should be no systematic differences in these ratings as a function of event type.

## The Present Study

In brief, the present study was designed to address two issues. First, we interested in comparing people's beliefs about script-consistent events with their beliefs about script-divergent events in the absence of direct event-relevant experience. Here we expected to replicate the standard differences in likelihood of occurrence, valence, and age normativity. We also expected to find that the two types of events would be quite similar in terms of their predicted importance and transitional impact. Second, we were interested in determining how experience affected the likelihood judgements and whether people's expectations about major life events and their experience with them aligned. Here we had grounds for predicting that experience would increase likelihood judgments, whereas the ratings for importance, impact and the valence might be less extreme in the experienced condition than the hypothetical condition.

## Method

### Participants

We recognized that older individuals were likely to have experienced more script-consistent and, importantly, more script-divergent events than younger people. Thus, to ensure enough responses from people who had and had not experienced the target events, we collected data from 188 younger adults (18–25 years, *M* = 18.95, *SD* = 1.21, 123 females) and 60 older adults (50–65 years, *M* = 57.02, *SD* = 4.28, 34 females). The younger adults were undergraduates enrolled in an introductory psychological course at the University of Alberta, and the older adults were recruited from local communities in Edmonton. Each younger participant received partial course credits, and each older participant received $15 as an honorarium. Participation was restricted to Canadians who had been born or lived in Canada for more than 20 years and spoke English as their first language. These restrictions were added to eliminate language and culture-related differences. All the younger participants received some postsecondary education; 17% of the older participants had secondary school diplomas or lower level of education, 63% received some postsecondary education or had graduated from college, and 20% had some postgraduate education or degrees. The participants were randomly assigned to two conditions. One older participant in the experienced condition withdrew and thus their data were excluded. At the end, there were 94 younger and 29 older participants in the experienced condition, and 94 younger and 30 older participants in the hypothetical condition.

### Materials

We selected 15 script-consistent events from Rubin et al.'s (2009) the set of life-script events nominated by the United States undergraduates, and 15 script-divergent events from Holmes and Rahe (1967) and Svob et al. (2014). The complete set of event descriptions is presented in the Appendix I.

We used the TIS-12 (Svob et al., 2014) to gauge the imagined (in the hypothetical condition) or assessed (in the experienced condition) transitional impact of the to-be-tested events. This scale consists of 12 items (see Table 1); 6 load on a material-impact subscale, and 6 on a psychological-impact subscale. The order of the statements was randomized for each participant. Participants indicated their agreement with each statement using a 1(*strongly disagree*)-to-5 (*strongly agree*) scale. Material-impact scores were calculated by averaging the ratings of the six material items and psychological-impact scores were averages of the psychological ratings.

**Table 1:** Transitional impact scale (TIS-12, Svob et al., 2014).

**Subscale**	**Item**
Material impact	This event has changed the places where I spend time.
	This event has changed the things I own.
	This event has changed my material circumstances.
	This event has changed the activities I engage in.
	This event has changed the people I spend time with.
	This event has changed where I live.
Psychological impact	This event has changed my attitudes.
	This event has changed the way I think about things.
	This event has impacted my emotional responses.
	This event has changed my sense of self.
	This event has impacted me psychologically.
	This event has influenced my understanding of right and wrong.

*In the hypothetical condition, the statements begin with “This event is likely to change…,” and use “person” and “the person's” in place of “I” and “my.”*

### Procedures

The stimulus presentation and data collection were programed with E-prime 2.0. All the phases were controlled by a computer and conducted in a laboratory. During Phase 1, all the participants were presented with the 30 event descriptions, one at a time, in a random order, and were asked whether they had experienced each. According to participants' responses, the computer program labeled each event as either “experienced” or “not experienced” (hypothetical), and randomly selected up to 8 script-consistent experienced (or hypothetical) events and up to 8 script-divergent experienced (or hypothetical) events, for a maximum total of up to 16 events. That is, if participants had endorsed more than 8 events for one event type (script-consistent or script-divergent) during Phase 1, they would only be re-presented with 8 in the subsequent phases; however, if participants had endorsed fewer than 8 of one event type, they would be re-presented all the endorsed items.

Following Phase 1, participants in the experienced condition were only presented with the events they indicated they had experienced, and those in the hypothetical condition were only presented with events that they had not experienced.

During Phase 2, experienced-condition participants provided a brief description for each event and estimated their own age at the time of the event. For example, one participant provided the following event description in response to the event *sustain a serious injury*: “Broke my arm when I was younger.” If a participant had experienced more than one event from a given category (e.g., marriage and remarriage), they were instructed to describe the first event in the series. During Phase 2, hypothetical-condition participants were asked to imagine “the life of an average Canadian who is about your age and who is also your gender,” and to estimate the likelihood (from 0 to 100%) that each event would occur in this average person's life. They were informed that an estimate *X* would be interpreted as “*X* out of 100 average Canadians would experience this event at some point during their lives.” Participants then indicated at what age (from 1 to 100 years) this average Canadian was most likely to undergo this event.

In Phase 3, in the hypothetical condition, the generic event descriptions were presented again, one at a time, in a random order. Participants were prompted to rate the likely importance (1 = *not important at all*; 5 = *very important*), emotional positivity (1 = *neutral*; 5 = *very positive*), emotional negativity (1 = *neutral*; 5 = *very negative*), and transitional impact (TIS-12, Svob et al., 2014) of each event from the perspective of that average Canadian. In the experienced condition, the event descriptions collected during Phase 2 were presented, one at a time, and participants rated each in terms of its *personal* importance, positivity, negativity, and transitional impact.

Finally, in a fourth phase, experienced-condition participants were asked to imagine “the life of an average Canadian who is about your age and who is also your gender.” Then, they provided likelihood-of-occurrence estimates (from 0 to 100%) and estimated age-of-occurrence estimates (from 1 to 100 years) for each of the generic event descriptions. The Phase 4 of the experienced condition was identical to the Phase 2 of the hypothetical condition.

## Results

In response to our two research questions—How do script-consistent events differ from script-divergent events, if they differ at all? How does experience affect people's beliefs about these life events?—we focus our analyses on the main effects of two factors: event type (script-consistent, script-divergent) and condition (experienced, hypothetical). As noted above, our primary motivation for including a group of older adults was to obtain information about the effect of experience on script-divergent events. We combine the data from the younger and older participants in order to obtain sufficient numbers of events in all four categories (experienced script-consistent, experienced script-divergent, hypothetical script-consistent, and hypothetical script-divergent)^2^. Below, we consider the following dependent variables: (a) prevalence of the target events in our sample, (b) estimated likelihood of occurrence of the events in an average Canadian's life, (c) estimated age at occurrence of the events in an average Canadian's life, (d) rated valence, and (e) importance and transitional impact ratings. We use non-parametric tests here (i.e., Mann-Whitney tests for two independent data sets) because, following Phase 1, each participant received a customized set of event descriptions. Individualized event selection depended on the condition the participant was assigned to and on the events that they had experienced. In addition, because script-consistent events were more likely to have been experienced than script-divergent events, participants in the experienced condition were, on average, presented with more script-consistent events (*M* = 6.67) than script-divergent events (*M* = 3.73), and participants in the hypothetical condition saw fewer script-consistent events (*M* = 6.68) than script-divergent events (*M* = 7.77). We also fitted a set of regression models to test the main effects of condition and event type and their interaction as fixed factors, with participant and event as random factors (when applicable). Results are presented in the Appendix II.

### Actual Prevalence of Events in Younger and Older Participants

**Figure 1:**
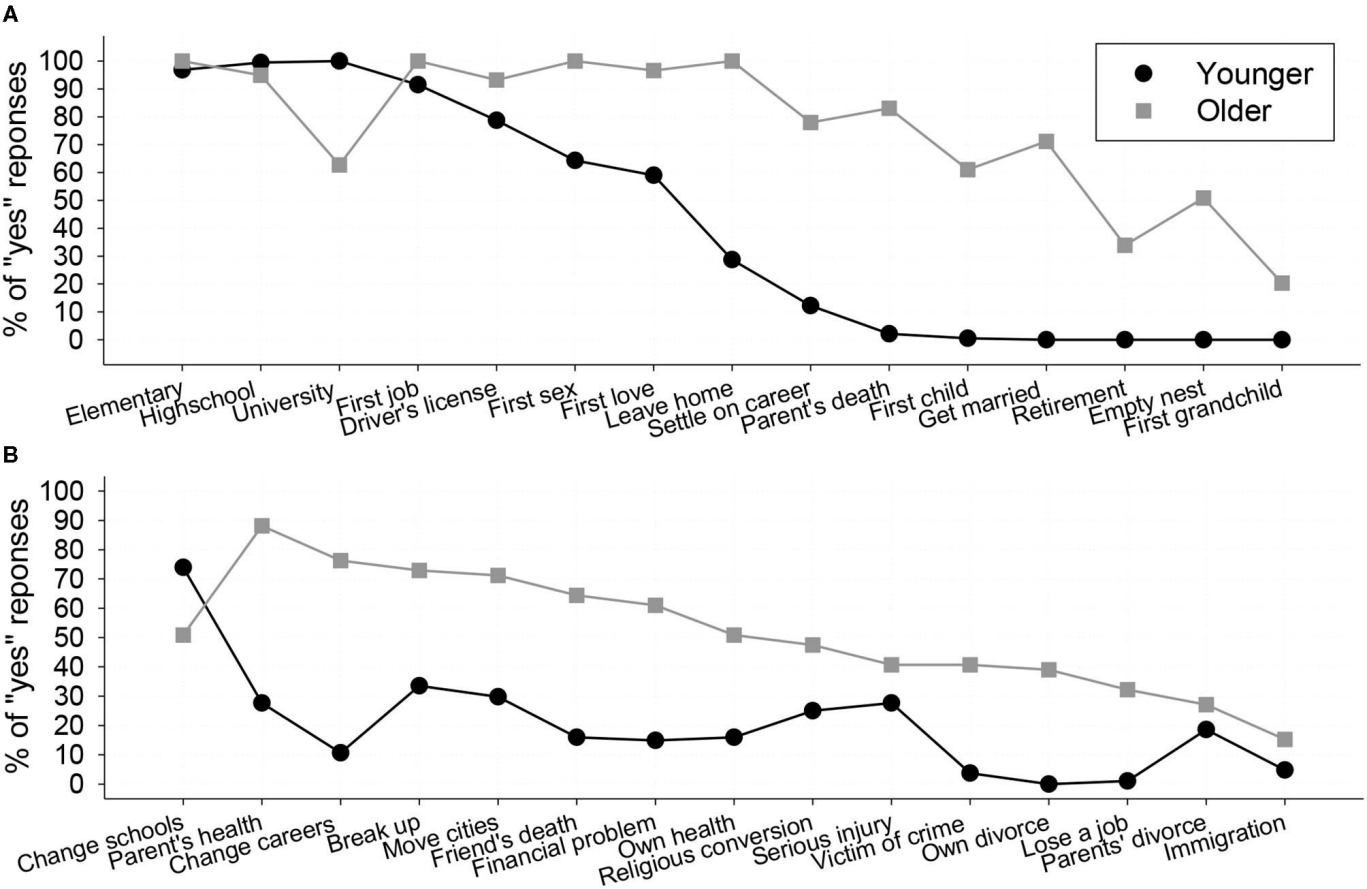
The prevalence of personally experienced script-consistent events **(A)** and script-divergent events **(B)** in the younger and older participants.

The primary goal of Phase 1 was to identify the events that were experienced by our participants, so that we could create individualized stimulus sets for the subsequent phases. However, these data are of interest on their own as they provide information about the prevalence of these events in the lives of our participants. Figure 1 presents the percentage of older and younger participants indicating that they had experienced the script-consistent events (top panel) and the script-divergent ones (bottom panel). On average, younger participants indicated that they had experienced 42.23% of the script-consistent events and 20.21% of the script-divergent ones; for the older participants, the comparable values were 76.38% and 51.86%. Overall, the script-consistent events were 2.92 times more likely to be experienced than the script-divergent ones, *B* = 1.07, *SE* = 0.05, Wald $\chi_{\left(1\right)}^{2}$ = 419.39, *p* < 0.001; older adults were 4.33 times more likely to indicate that they had experienced an event than younger adults, *B* = 1.47, *SE* = 0.06, Wald $\chi_{\left(1\right)}^{2}$ = 599.67, *p* < 0.001.

### Comparing Script-Consistent and Script-Divergent Events

#### Predicted Likelihood of Occurrence

**Figure 2:**
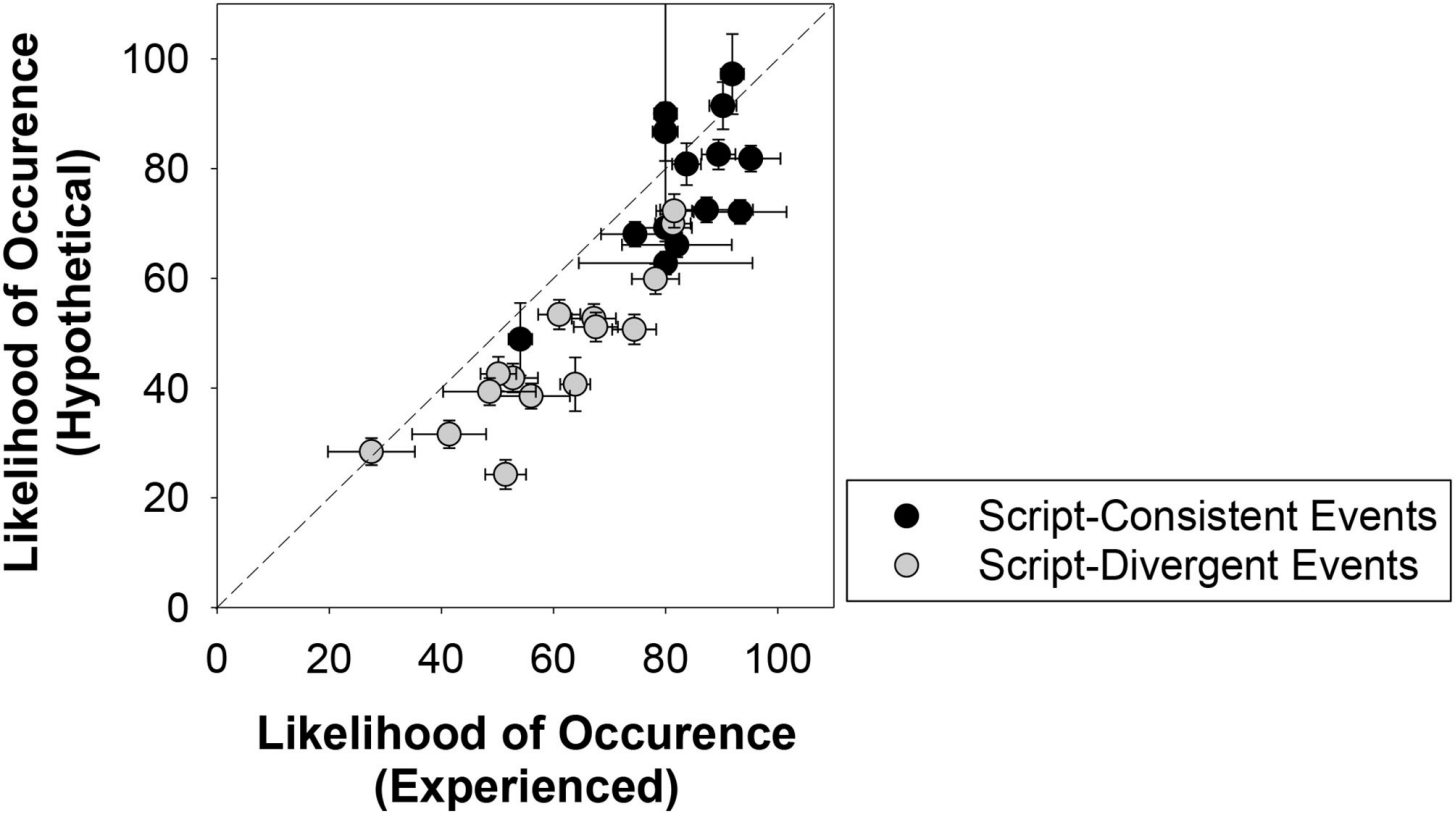
Mean likelihood-of-occurrence of script-consistent and script-divergent events in an average Canadian's life produced by participants in the experienced and hypothetical conditions. Error bars indicate ± 1 standard error of mean. The event, “begin grade school,” is omitted in this graph because it only has estimates from the experienced condition (*M* = 95.04, *SE* = 2.09).

Figure 2 presents the predictions about how likely each event would occur in an average Canadian's life produced by the participants in the experienced and hypothetical conditions. Consistent with prior findings, the likelihood estimates were higher for script-consistent events than for the script-divergent events; averaging over conditions, script-consistent events received a mean likelihood of 80.24% (*SD* = 12.18) and the script-divergent events received a mean likelihood of 53.34% (*SD* = 16.09). Mann-Whitney tests showed that the differences in the likelihood-of-occurrence between script-consistent and script-divergent events were significant for both the experienced condition, *U*_(15, 15)_ = 20.00, *z* = −3.84, *p* < 0.001 (two-tailed), effect size *r* = 0.70, and the hypothetical condition, *U*_(14, 15)_ = 16.00, *z* = −3.88, *p* < 0.001 (two-tailed), effect size *r* = 0.72. Put it another way, in general, participants believed that the events that could be mapped onto a life script were more likely to occur than the events could not.

#### Age Normativity

**Figure 3:**
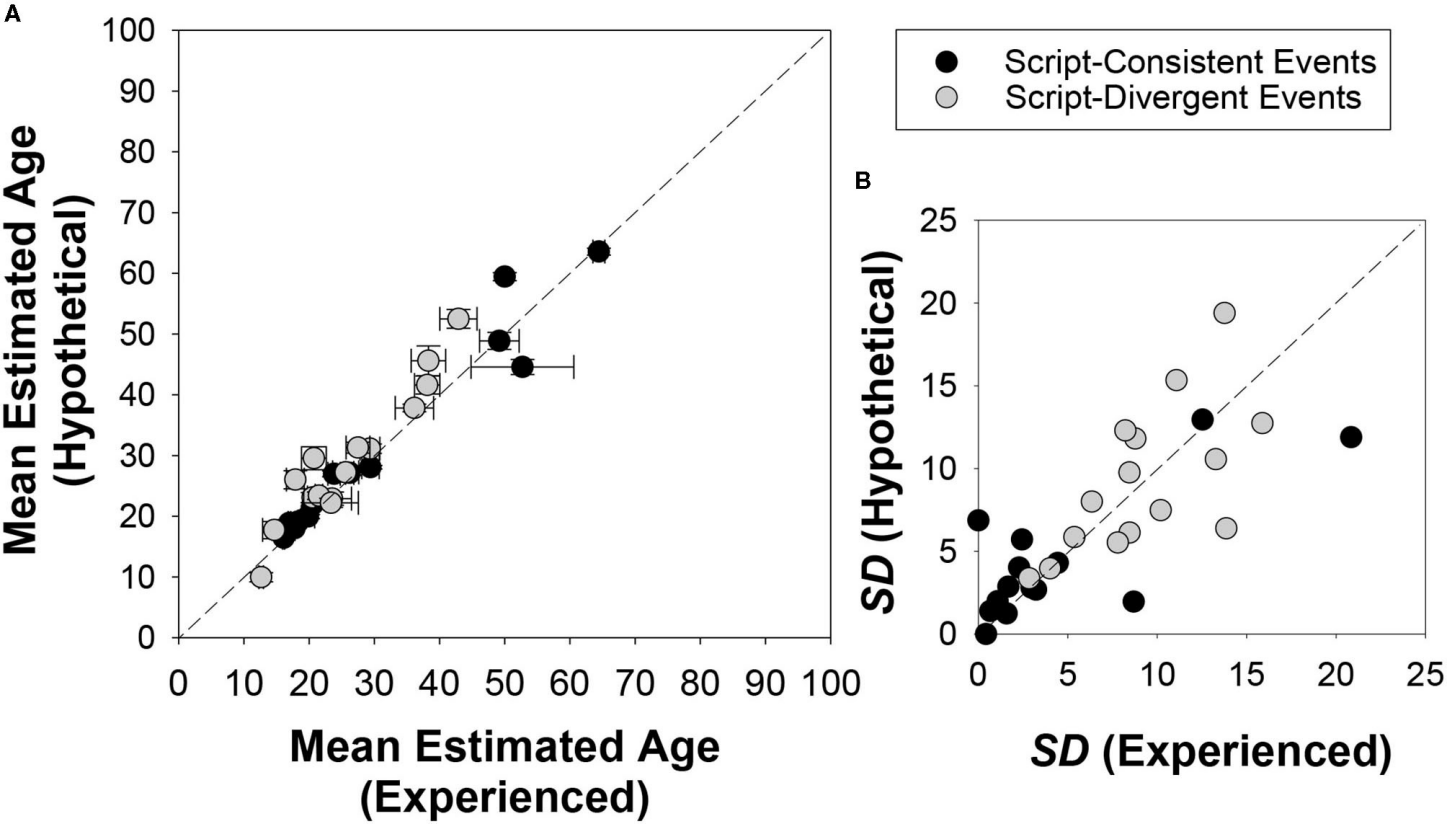
Mean **(A)** and SD **(B)** of the estimated age of an average canadian at the occurrence of the events produced by participants in the experienced and hypothetical conditions. Error bars indicate ± 1 standard error of mean. The event, “begin grade school,” is omitted in both graphs because it only has estimates from the experienced condition (*M* = 5.68, *SD* = 1.39, *SE* = 0.13).

To investigate age normativity, we calculated the means and standard deviations of the age estimates provided for each event (across participants) in the two conditions. Results are shown in Figure 3. Consistent with prior research, we found that average deviation was smaller for the script-consistent events (*M* = 4.46, *SD* = 4.82) than for the script-divergent events (*M* = 9.23, *SD* = 4.08). Mann-Whitney tests suggested that the script-consistent events were believed to be more temporally prescribed than the script-divergent events by the participants in the experienced condition, *U*_(15, 15)_ = 38.00, *z* = −3.09, *p* = 0.001 (two-tailed), effect size *r* = 0.56, and those in the hypothetical condition, *U*_(13, 15)_ = 37.00, *z* = −2.79, *p* = 0.004 (two-tailed), effect size *r* = 0.53.

#### Valence

**Figure 4:**
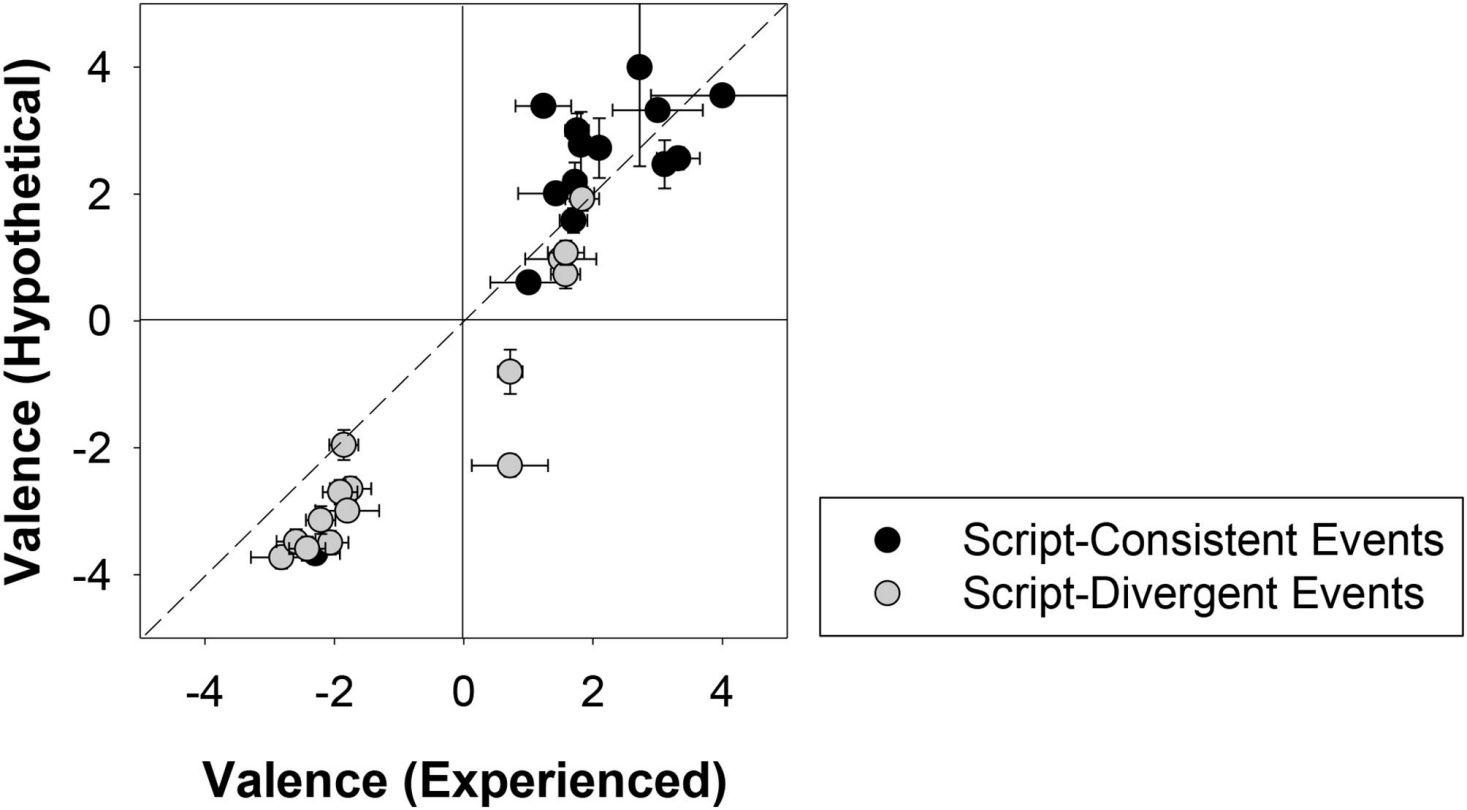
Mean ratings of the valence of script-consistent events and script-divergent events in the experienced condition (participants' real life) and the hypothetical condition (an average Canadian's life). Error bars indicate ± 1 standard error of mean. The event, “begin grade school,” is omitted in this graph because it only has estimates from the experienced condition (*M* = 2.10, *SE* = 0.15).

To calculate the average valence, we first subtracted each participant's negative emotional rating for a given event from their positive emotional rating for that same event. Thus, a positive value indicates that participants tended to believe it was a positive event (in their real life or in an average Canadian's life), and a negative value indicates the opposite. Events with a value near zero were either affectively neutral or associated with complex emotions. Figure 4 shows a dissociation of script-consistent events and script-divergent events in the measure of valence. Consistent with our prediction and much prior research (Erdogan et al., 2008; Dickson et al., 2011; Thomsen et al., 2011; Umanath and Berntsen, 2013; Grysman and Dimakis, 2018; Janssen and Haque, 2018), script-consistent events were generally positive (*M* = 2.04, *SD* = 1.65), whereas the script-divergent events tended to be negative (*M* = −1.25, *SD* = 1.93). Mann-Whitney tests yielded significant differences in the rated valence between script-consistent and script-divergent events for both the experienced condition, *U*_(15, 15)_ = 28.00, *z* = −3.50, *p* < 0.001 (two-tailed), effect size *r* = 0.64, and the hypothetical condition, *U*_(14, 15)_ = 19.00, *z* = −3.75, *p* < 0.001 (two-tailed), effect size *r* = 0.70.

#### Importance and Transitional Impact

**Figure 5:**
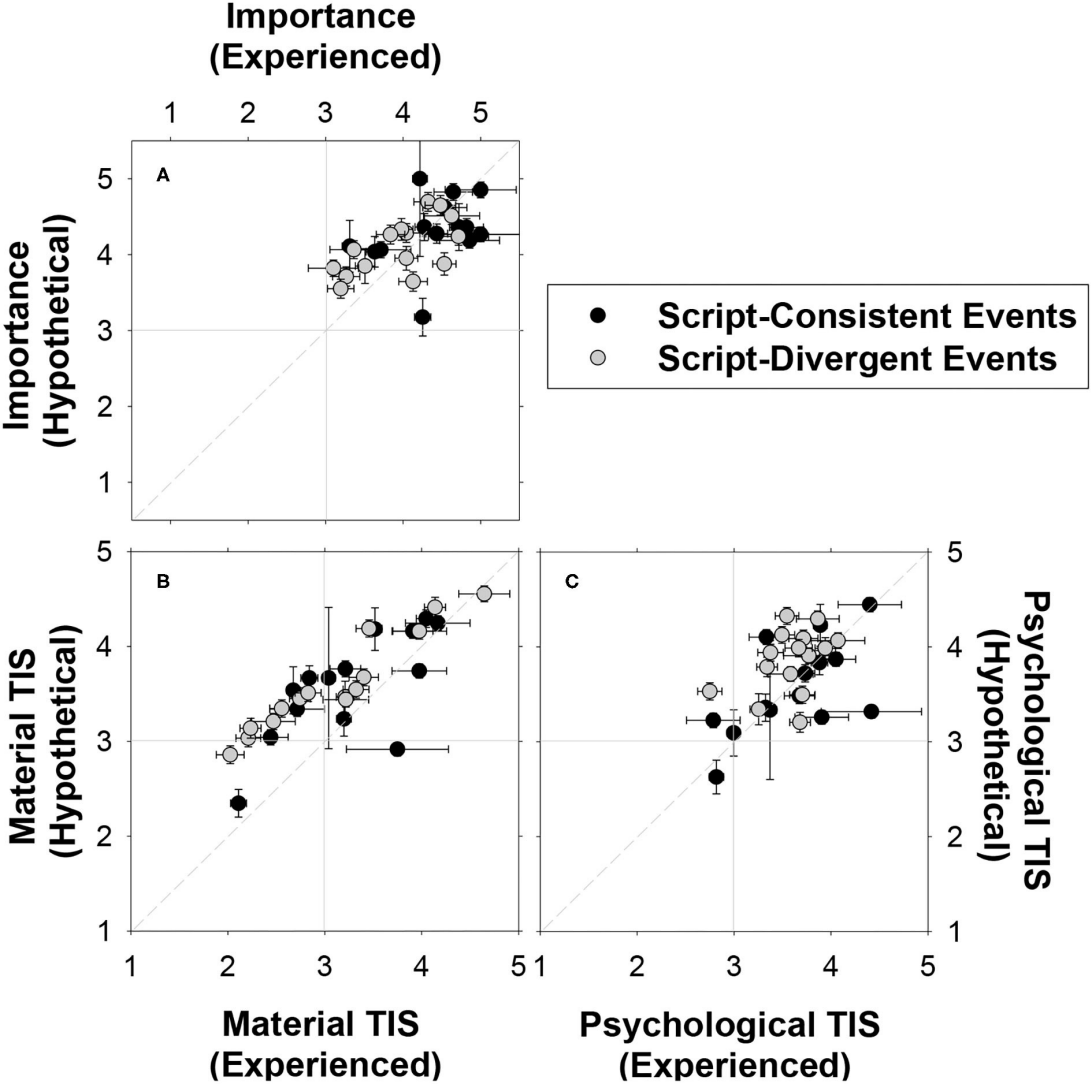
Mean ratings of the importance **(A)**, material impact **(B)** and psychological impact **(C)** of script-consistent events and script-divergent events in the experienced condition (participants' real life) and the hypothetical condition (average Canadian's life). Error bars indicate ± 1 standard error of mean. The event, “begin grade school,” is omitted in the graphs because it only has estimates from the experienced condition (mean importance = 3.48, *SE* = 0.10; mean material TIS score = 2.66, *SE* = 0.07; mean psychological TIS score = 3.30, *SE* = 0.07).

Previously we have predicted that both script-consistent and script-divergent events would often be considered as important life transitions and that differences that might exist are likely to be small. As shown in Figure 5, both types of events received moderate to high ratings for importance (*M*_consistent_ = 4.32, *SD*_consistent_ = 0.50; *M*_divergent_ = 4.02, *SD*_divergent_ = 0.46), material impact (*M*_consistent_ = 3.39, *SD*_consistent_ = 0.63; *M*_divergent_ = 3.35, *SD*_divergent_ = 0.69), and psychological impact (*M*_consistent_ = 3.58, *SD*_consistent_ = 0.50; *M*_divergent_ = 3.72, *SD*_divergent_ = 0.35). We conducted Mann-Whitney tests separately for the experienced and hypothetical conditions. Results indicated that the observed differences between the script-consistent events and script-divergent events were not statistically significant for the material and psychological TIS scores in both conditions as well as for the importance ratings in the hypothetical condition (all *p* > 0.05, effect sizes *r* = [0.01, 0.32]). Participants in the experienced condition indicated that the script-consistent events (*M* = 4.33, *SD* = 0.56) were more important than the script-divergent events (*M* = 3.94, *SD* = 0.55), Mann-Whitney *U*_(15, 15)_ = 65.00, *z* = −1.97, *p* = 0.049 (two-tailed), effect size *r* = 0.36. Despite this small difference, script-consistent and script-divergent events are considered to be roughly comparable in terms of their overall importance and their transitional impact.

### Comparing Experience With Hypothetical Beliefs

#### Event Likelihood, Importance, and Impact

The second goal of the present research was to assess the ways that beliefs about life transitions are affected by experience. To investigate this issue, we collapsed across script-consistent and script-divergent events and conducted a series of Mann-Whitney tests on the differences between the experienced condition and the hypothetical condition.

Among the results, two things stand out. First, as predicted, participants in the experienced condition (*M* = 71.98, *SD* = 17.67) provided higher likelihood of occurrence estimates than participants in the hypothetical condition (*M* = 60.95, *SD* = 20.26). A Mann-Whitney test confirmed this observation, *U*_(30, 29)_ = 296.00, *z* = −2.11, *p* = 0.035 (two-tailed), effect size *r* = 0.27. The results suggest that when people have experienced an event, they tend to believe it is more common (i.e., an availability bias). Second, material TIS scores were higher in the hypothetical conditions (*M* = 3.59, *SD* = 0.53) than in the experienced condition (*M* = 3.16, *SD* = 0.70), Mann-Whitney *U*_(30, 29)_ = 261.50, *z* = −2.63, *p* = 0.008 (two-tailed), effect size *r* = 0.34. This, however, was not true for the importance ratings (*M*_experienced_ = 4.13, *SD*_experienced_ = 0.58; *M*_hypothetical_ = 4.21, *SD*_hypothetical_ = 0.41, *U*_(30, 29)_ = 417.00, *z* = −0.27, *p* = 0.789, effect size *r* = 0.04) or the psychological TIS scores (*M*_experienced_ = 3.59, *SD*_experienced_ = 0.42; *M*_hypothetical_ = 3.71, *SD*_hypothetical_ = 0.44, *U*_(30, 29)_ = 359.50, *z* = −1.14, *p* = 0.256, effect size *r* = 0.15).

#### Valence

Overall, the valence scores did not differ as a function of experience, *M*_experienced_ = 0.57, *SD*_experienced_ = 2.11; *M*_hypothetical_ = 0.15, *SD*_hypothetical_ = 2.75, *U*_(30, 29)_ = 402.00, *z* = −0.50, *p* = 0.622 (two-tailed), effect size *r* = 0.07. Figure 4 suggests that valence judgments might be more extreme in the hypothetical conditions than in the experienced condition. However, no reliable difference was found between conditions for script-consistent events, *M*_experienced_ = 1.91, *SD*_experienced_ = 1.44; *M*_hypothetical_ = 2.18, *SD*_hypothetical_ = 1.89, *U*_(15, 14)_ = 77.50, *z* = −1.20, *p* = 0.230 (two-tailed), effect size *r* = 0.22, and the difference was only marginally significant for script-divergent events, *M*_experienced_ = −0.77, *SD*_experienced_ = 1.81; *M*_hypothetical_ = −1.74, *SD*_hypothetical_ = 1.98, *U*_(15, 15)_ = 67.00, *z* = −1.89, *p* = 0.059 (two-tailed), effect size *r* = 0.34. These results, along with the previous valence analyses, indicated that the script-consistent events tended to be positive whereas the script-divergent events tended to be negative, regardless of whether they were experienced.

#### Age Normativity

Janssen and Rubin (2011) pointed out that participants' age estimates reflected their semantic knowledge about a normative life script, rather than the episodic memories of their own experiences. To examine the possible effect of experience on the predicted age, we plotted the estimates obtained in the hypothetical condition as a function of those of the experienced condition. As shown in Figure 3 (left panel), the predicted age at event in the two conditions were highly correlated for both the script-consistent events, *r*_(14)_ = 0.98, *p* < 0.001, and the script-divergent events, *r*_(15)_ = 0.95, *p* < 0.001. Figure 3 (right panel) also presents the standard deviation scores. Collapsing across event type, the average deviation score was 6.75 years (*SD* = 5.37) for the experienced condition and 7.11 years (*SD* = 4.72) for the hypothetical condition. The Mann-Whitney test indicated that this between-condition difference was not statistically significant, *U*_(30, 28)_ = 392.00, *z* = −0.44, *p* = 0.671 (two-tailed), effect size *r* = 0.06. Consistent with Janssen and Rubin (2011), personal experience had little influence on beliefs about the normative age of the events.

#### Between-Group Correlations

One final point. Although experience affected the measures of likelihood and material impact as we predicted, the data presented in Figure 2 through Figure 5 make it clear that, in general, ratings, and judgments provided in the experienced and hypothetical conditions were very similar in an ordinal sense. Specifically, for likelihood-of-occurrence, the rank-order correlation between the judgments provided in the experienced and hypothetical conditions was very high [*r*_(29)_ = 0.89, *p* < 0.001] as were the correlations for material impact [*r*_(29)_ = 0.84, *p* < 0.001], valence [*r*_(29)_ = 0.91, *p* < 0.001], and to a lesser degree, importance [*r*_(29)_ = 0.59, *p* = 0.001], and psychological impact [*r*_(29)_ = 0.39, *p* = 0.035].

## General Discussion

This study was undertaken with two aims in mind. First, we were interested in comparing people beliefs about script-consistent and script-divergent events in order to assess their similarities and their differences. Second, we were interested in determining whether experience affects beliefs about these events. Regarding the first issue, we found that, as expected and consistent with prior research: (a) script-consistent events were considered more likely than script-divergent event, (b) age estimates provided for script-consistent events were less variable than those provided for script-divergent events, and (c) script-consistent events were considered to be more positive than script-divergent events (Greene, 1990; Greene et al., 1992; Settersten and Hagestad, 1996a,b; Berntsen and Rubin, 2004; Habermas, 2007; Erdogan et al., 2008; Rubin et al., 2009; Dickson et al., 2011; Thomsen et al., 2011; Umanath and Berntsen, 2013; Alea et al., 2014; Bohn and Habermas, 2016; Enz and Talarico, 2016; Grysman and Dimakis, 2018; Janssen and Haque, 2018). We also found that (d) the two types of events differed little in terms of material change, psychological change, or rated importance. This point is driven home by data presented in Figure 5, which demonstrates that participants recognize that most of events they were presented with, regardless of whether the events were script-consistent or script-divergent, have the potential of producing considerable change in a person's life. More generally, these findings suggest that the script-consistent events sampled here and the script-divergent ones are members of same category, the category of potentially important life events. It is true that people are aware that restrictive age norms are associated with some events and that some events are more likely to occur than others are. However, it is also clear that people hold beliefs about the importance and possible impact of potentially life-changing events, and that these beliefs, expressed by participants in the hypothetical condition, are not strongly related to an event's status as a member of the cultural life script.

With regard to the second issue, we observed several between-group differences. Specifically, we found that as events were rated as being (e) more likely when they were experienced directly than when they considered prospectively, a finding consistent with prior research indicating that availability can influence likelihood judgements (Tversky and Kahneman, 1973; Carroll, 1978; Lichtenstein et al., 1978; Weinstein, 1980; Fitzgerald et al., 1988; MacLeod and Campbell, 1992; Brown, 2002). We also found that compared to the ratings provided by participants in the hypothetical condition, individuals with event-relevant lived experience tended to consider these events to have had produced less material change. This finding can be interpreted in two ways. One possibility is that our experiences typically fail to live up to our expectations (e.g., Lauterbach and Vielhaber, 1966; Stern, 1966; Buckley, 1971; King and Walsh, 1972; Belsky, 1985; Luhmann et al., 2012). A second is that our memory for these experiences and their repercussions fades over time (e.g., Barclay and Wellman, 1986; Wagenaar, 1986; Lindeman et al., 2017).

Despite these minor differences, it is fair to say that we found a close correspondence between the data provided in the experienced condition and those provided in the hypothetical condition. From this, we conclude that people have a fairly accurate understanding of the likely outcome of various important life transitions even when they have not lived through them. This claim is important from a methodological perspective because it suggests that researchers need not be overly concerned about with the presence of experienced individuals in a sample. It is also important in a practical sense; broadly speaking, people appear to have a realistic, if imperfect, sense of what to expect when they are confronted with (or choose to undertake) a major change in their lives.

In brief, script-consistent events differ from script-divergent events in several ways; compared to script-divergent events, script-consistent events are considered to be more likely, more temporally normative, and more positively. However, events from the two categories are very similar in terms of importance and transitional impact. This pattern of similarities and dissimilarities raises an interesting issue. Do we need to distinguish between script-consistent and script-divergent events, or can we treat events of both types as members of a larger category, the category of common, potentially important life transitions? We conclude by arguing that distinction is useful in some contexts and unnecessary in others.

First, consider the role of important life events in autobiographical memory. Prior research has demonstrated that script-consistent and script-divergent events organize autobiographical memory and spawn memorable experiences. As noted in the introduction, both types of events serve as temporal landmarks (e.g., Bohn and Habermas, 2016; Shi and Brown, 2016; Gu et al., 2017; Camia et al., 2019), can give rise to “bumps” in the temporal distributions of recalled events (e.g., Pillemer et al., 1986, 1988; Kurbat et al., 1998; Thomsen and Berntsen, 2005; Uzer and Brown, 2015; Brown et al., 2016; Enz et al., 2016; Shi and Brown, 2016; Gu et al., 2017; Thomsen et al., 2021) and are mentioned when people narrate their life stories (Glück and Bluck, 2007; Thomsen and Berntsen, 2008; Rubin et al., 2009; Bohn, 2010; Haque and Hasking, 2010; Dickson et al., 2011; Thomsen et al., 2011; Gu et al., 2017, 2019) and when adult children recall their parents' lives (Svob and Brown, 2012; Svob et al., 2016; Gu et al., 2019). Transition Theory (Brown et al., 2012, 2016; Brown, 2016, 2021) contends that these phenomena reflect the degree of change engendered by the transitions in question. Given that script status is not strongly related to experienced impact (Figure 5), it follows that, at least from a memory perspective, there appears little need to distinguish script-consistent events from script-divergent ones.

This conclusion is not intended to deny the psychological reality of the culture life script. Certainly, there is a large and growing body of evidence indicating that life scripts of the sort defined by Berntsen and Rubin (2004) can be found in every culture (Erdogan et al., 2008; Rubin et al., 2009; Zaragoza Scherman, 2013; Janssen et al., 2014; Zaragoza Scherman et al., 2017; Janssen and Haque, 2018). It also appears to be true that the life scripts are used in plotting out and assessing life trajectories, i.e., remembering one's past and predicting one's future (Hareven and Masoka, 1988; Greene, 1990; Greene et al., 1992; Schrauf and Rubin, 2001; Collins et al., 2007; Habermas, 2007; Berntsen and Jacobsen, 2008; Bohn and Berntsen, 2008, 2011, 2013; Thomsen and Berntsen, 2008; Berntsen and Bohn, 2010; Thomsen et al., 2011; Grysman et al., 2015; Enz et al., 2016; Ramsgaard and Bohn, 2021). There is also evidence that people experience distress when they fail to meet the milestones established by life scripts (Kuwabara et al., 2007; Rubin et al., 2009; Shanahan and Busseri, 2016; Dunlop et al., 2017). Moreover, the life-script research is useful in determining how social norms and expectations change over time and how they differ between sub-populations within a given society (Bohn, 2010; Janssen and Rubin, 2011; Janssen et al., 2014). Taken together, these findings make it clear that a cultural life script represents an important form of shared autobiographically relevant semantic knowledge. The present study extends this idea by demonstrating that people hold shared beliefs about script-divergent events as well.

## Data Availability Statement

The original contributions presented in the study are included in the article/Supplementary Material, further inquiries can be directed to the corresponding authors.

## Ethics Statement

The studies involving human participants were reviewed and approved by Research Ethics Boards at University of Alberta. The patients/participants provided their written informed consent to participate in this study.

## Author Contributions

LS and NB designed the experiment and supervised the data collection. LS programmed the experiment, performed all data analyses, and wrote the first draft of the manuscript. NB supervised the research process and provided critical revisions on the manuscript. All authors contributed to the article and approved the submitted version.

## Conflict of Interest

The authors declare that the research was conducted in the absence of any commercial or financial relationships that could be construed as a potential conflict of interest.

## Publisher's Note

All claims expressed in this article are solely those of the authors and do not necessarily represent those of their affiliated organizations, or those of the publisher, the editors and the reviewers. Any product that may be evaluated in this article, or claim that may be made by its manufacturer, is not guaranteed or endorsed by the publisher.

## Footnotes

^1^It is also true people know that certain script-divergent events can be highly probable (e.g., major illness, relocation), but they also appear to understand that such events can happen at any time, which means that they are, by definition, excluded from the set of events that make up the cultural life-script.

^2^We acknowledge the limitation of combining data from the two different age groups. For example, people's attitudes toward a “typical” life script may change as they age (Bohn, 2010; Tekcan et al., 2012) and younger adults may still be in the process of developing their life-script knowledge (Habermas, 2007). However, it is impossible to obtain equal numbers of experienced and unexperienced script-consistent/divergent events with only one age group. As a result, we resolve the problem using this non-conventional method.
