# Supplementary material for: Beliefs About Transitional Events: The Effect of Experience and Life-Script Consistency
Source: Front Psychol. 2021 Aug 11;12:727524. doi: 10.3389/fpsyg.2021.727524 (PMC8385369; doi:10.3389/fpsyg.2021.727524)
Supplement: Supplementary file 2 [file Data_Sheet_1.pdf]

Appendix I: Sources of Events

Present study	Rubin et al. (2009)	Holmes & Rahe (1967)	Svob et al. (2014)
**Script-consistent events**			
Begin grade school	Begin school	Begin school	
Graduate from high school	High school	End school	
Begin university	College		Starting university
Begin first serious romantic relationship	Fall in love		
Get first job	First job		
Have first sexual experience	First sex, first kiss		
Leave parent's home	Leave home	Change in residence	
Get married	Marriage	Marriage	
Birth of the first child	Having children	Pregnancy; Gain of new family member	
Obtain driver's license	Begin driving		Getting a car
Settle on career	Settle on career		
Begin retirement	Retirement	Retirement	
Death of a parent	Parents' death		
Have children move out of home	Empty nest	Son or daughter leaving home	
Birth of first grandchild	Grandchildren	Gain of new family member	
**Script-divergent events**			
Parent divorce one another			Parents' divorce
Immigrate to a new country			Immigrating to Canada
Move to a distant city		Change in residence	Moving from one city to another
End a serious romantic relationship			Break-up of a serious romantic relationship
Sustain a serious injury		Personal injury	Sustaining a serious injury
Be diagnosed with a serious health problem		Personal illness	Being diagnosed with a serious health problem
Be a victim of a criminal assault			Being a victim of a criminal assault
Experience a religious conversion		Change in church activities	Religious conversion
Change to a new school		Change in schools	
Change careers		Change to different line of work	
Be fired from a full-time job		Fired at work	
Experience the death of a close friend		Death of close friend	
Deal with health problems of an aging parent		Change in the health of family member	
Undergo a serious financial problem		Change in financial state	
Get divorced		Divorce	

Appendix II: Regression Results

Mean (And SD) Ratings for the Likelihood-Of-Occurrence, Age Normativity, Valence, Importance, and Transitional Impact of Script-Consistent and Script-Divergent Events Given by Participants in the Experienced and Hypothetical Conditions

	Main Effects						Interaction				
	Condition			Event Type			Script-consistent		Script-divergent		*p*
	Experienced	Hypothetical	*p*	Script-consistent	Script-divergent	*p*	Experienced	Hypothetical	Experienced	Hypothetical	
Likelihood[a]	76.23 (22.95)	57.78 (30.15)	***	77.58 (24.37)	51.48 (27.24)	***	82.64 (18.27)	72.53 (28.34)	64.78 (25.85)	45.10 (25.55)	***
Age Normativity[b]	6.99 (5.31)	7.11 (4.72)	ns	4.62 (4.82)	9.23 (4.08)	***	4.59 (5.76)	4.66 (3.82)	9.22 (3.81)	9.24 (4.48)	ns
Valence[c]	1.10 (2.63)	-0.11 (3.00)	***	1.98 (2.25)	-1.45 (2.48)	***	2.09 (2.06)	1.88 (2.41)	-0.66 (2.62)	-1.83 (2.31)	***
Importance[c]	4.01 (1.23)	4.24 (0.99)	+	4.22 (1.05)	4.05 (1.17)	***	4.06 (1.18)	4.39 (0.86)	3.90 (1.31)	4.12 (1.09)	ns
TIS – Material[c]	2.99 (1.04)	3.61 (0.81)	***	3.30 (0.96)	3.41 (0.96)	ns	2.99 (0.97)	3.61 (0.85)	2.99 (1.15)	3.62 (0.78)	ns
TIS – Psychological[c]	3.44 (0.95)	3.78 (0.71)	***	3.52 (0.84)	3.77 (0.81)	***	3.40 (0.93)	3.64 (0.73)	3.51 (0.98)	3.89 (0.67)	**

[a]significance levels determined by an event type (script-consistent, script divergent) × condition (experienced, hypothetical) LME, with participant and event as covariates.
[b]significance levels determined by an event type (script-consistent, script divergent) × condition (experienced, hypothetical) ANOVA.
[c]significance levels determined by an event type (script-consistent, script divergent) × condition (experienced, hypothetical) Ordinal Regression, with participant and event as covariates.
Ns = non-significant; + = $p < .10$; * = $p < .05$; ** = $p < .01$; *** = $p < .001$
